# Osteogenesis and aging: lessons from mesenchymal stem cells

**DOI:** 10.1186/s13287-018-0995-x

**Published:** 2018-09-26

**Authors:** Arantza Infante, Clara I. Rodríguez

**Affiliations:** Stem Cells and Cell Therapy Laboratory, BioCruces Bizkaia Health Research Institute, Cruces University Hospital, 48903 Barakaldo, Spain

**Keywords:** Mesenchymal stem cells, Osteogenesis, Aging, Differentiation shift

## Abstract

Aging is a high risk factor for the development of osteoporosis, a multifactorial age-related progressive disease characterized by reduced bone mass and increased risk of fractures. At the cellular level, the mesenchymal stem cell pool in the bone marrow niche shows a biased differentiation into adipogenesis at the cost of osteogenesis. This differentiation shift leads to decreased bone formation, contributing to the etiology of osteoporosis. This review will focus on the most recent/relevant molecular findings driving this functional impairment of mesenchymal stem cells in the aging process.

## Background

Aging is a gradual process that results in a loss of tissue homeostasis, driving a progressive deterioration of tissue and organ functions mainly due to cellular damage accumulated throughout life [1]. The human skeleton is especially affected by the passage of time: bone loss begins as early as the third decade of life, immediately after peak bone mass.

In humans, bone is a highly active tissue which undergoes continuous self-regeneration throughout adulthood to maintain structural integrity in a process called bone remodeling. It has been estimated that the entire skeleton is remodeled every 10 years [2]. This bone turnover comprises the temporal and spatial coordination of two processes performed in bone remodeling units at discrete sites throughout the skeleton: removal of old and damaged bone by osteoclasts, called resorption, which can last 4–6 weeks, followed by new bone formation by osteoblasts, which produce collagen and mineralized bone matrix in a process lasting 4–5 months [3]. Throughout young adulthood more bone is formed than is resorbed, resulting in an increase in bone mass. Later on, throughout adulthood when the growth period is finished, the amount of resorbed bone equals that which is subsequently formed (remodeling balance). In the elderly, the amount of bone resorbed is greater than the amount of bone formed; accordingly, a decrease in bone mineral density occurs. As a consequence, bone aging is the main risk factor for primary osteoporosis, characterized by a reduction in bone mineral density, predisposing the elderly population to an increased risk of fractures.

## Components of bone

Bone is a very dynamic and heterogeneous tissue formed by two components which are in close relationship with each other during the bone remodeling process: extracellular matrix (ECM) and bone cells. ECM is composed of an organic phase (20–40%), a mineral phase formed by hydroxyapatite crystals (50–70%) which confers rigidity and strength to bone, water (5–10%), and lipids (< 3%). The organic phase is mainly formed by collagen type I fibers, which provide elasticity and flexibility to bone, but also non-collagenous proteins as well, such as cytokines, growth factors, and proteoglycans. The latter interact with the cytokines and growth factors, regulating their function [4]. The bone remodeling process is orchestrated by different cell types originating from different progenitors, mesenchymal or hematopoietic precursors:

1. Mesenchymal stem cells (MSCs), which are activated by secreted, active transforming growth factor beta 1 (TGFβ) to migrate to bone-resorptive sites and differentiate into osteoblasts (see below).
2. Pre-osteoblasts, a heterogeneous population of cells, including those transitioning from MSCs to mature osteoblasts, which express the transcription factor runt related transcription factor 2 (RUNX2)*,* a key player in the osteogenesis process.
3. Osteoblasts, polarized cuboidal cells specialized for the active secretion of ECM. These cells have a relatively short-lifespan, estimated at 3 months in human bones [5]. The ECM, very rich in type I collagen, is known as the “osteoid” when first deposited and not yet mineralized. Mineralization occurs through the accumulation of calcium phosphate in the form of hydroxyapatite. Formation of mineralized ECM results in the hard but lightweight material that forms bone.
4. Osteocytes, which are the most abundant cells in bone, composing 90–95% of all bone cells in an adult. They are derived from terminally differentiated osteoblasts surrounded by unmineralized matrix (osteoid) during bone formation. Once the osteoid mineralizes, the osteocytes are trapped there and form an extensive network with each other, with osteoblasts, and with the lining cells on the bone surface (explained below). Contrary to osteoblasts, osteocytes can survive throughout the life of an individual [6]. As a feature, these cells have a small cell body and show numerous long, dendritic-like cytoplasmic prolongations that form a canalicular system inside bone [7]. They are the major mechanosensitive skeletal cell type and have critical roles in the regulation of osteoblast and osteoclast differentiation and function [8].
5. Bone lining cells (BLCs), post-mitotic, long-lived flat osteoblast lineage cells lining the bone surface. It was thought that their main function was to remove demineralized matrix on the bone surface before bone formation [9]. However, recent studies have pointed to a role for BLCs in bone remodeling, suggesting that, at least in adult mice, BLCs can be a source of osteoblasts in response to anabolic stimuli as well as under normal non pathological bone remodeling [10, 11].
6. Osteoclasts are, on the other hand, derived from monocyte-macrophage lineage cells. These multinucleated cells resorb bone by releasing enzymes which are active at a low pH, digesting proteins and releasing their fragments. After osteoclasts complete resorption, they undergo apoptosis.

## MSC osteogenic differentiation in health and aging

**Fig. 1:**
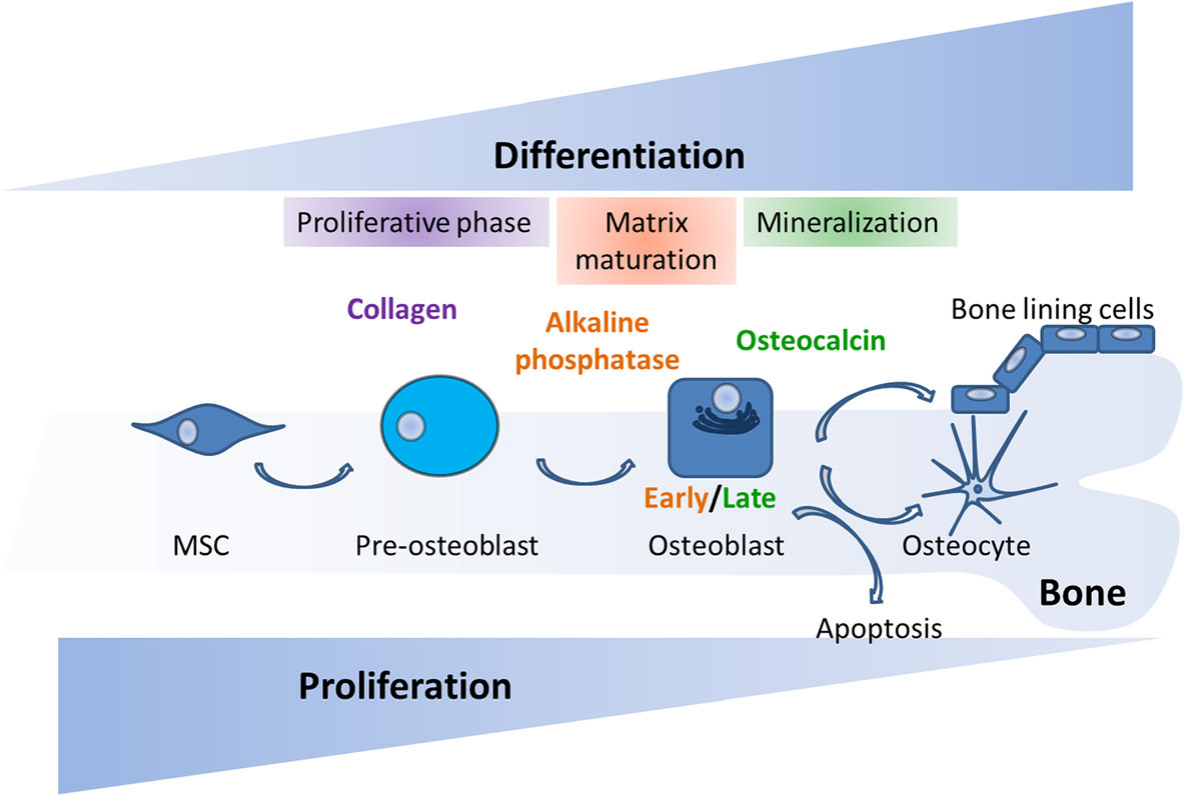
Osteogenic differentiation of MSCs. The MSC population proliferates actively at the initial stages of osteogenesis. As MSCs commit to osteoblasts their proliferation rate decreases while they start expressing osteogenic markers such as alkaline phosphatase secreted by early osteoblasts (matrix maturation phase) and osteocalcin secreted by late osteoblasts (mineralization phase). At the end of the bone forming phase, they can become BLCs or osteocytes or undergo apoptosis

MSCs are spindle shaped, adherent, non-hematopoietic stem cells which can be isolated from many tissues and have the capacity of self-renewal and to differentiate into various mesodermal cell types, such as osteoblasts, chondrocytes, and adipocytes [12]. In bone, the process of osteogenesis is driven by a sequential cascade of biological processes initiated by the recruitment of MSCs to bone remodeling sites and subsequent proliferation, lineage commitment, expression of lineage-specific markers, collagen secretion, and ECM mineralization [13]. During the first steps of differentiation, MSCs proliferate and commit to actively proliferating pre-osteoblasts which do not secrete ECM. They further mature into non-proliferating osteoblasts involved in initial matrix secretion, maturation, and mineralization. Once ECM is formed, osteoblasts have three possible fates: become osteocytes embedded in mineralized bone matrix and lose most of their cytoplasmic organelles; die by apoptosis; or become inactive quiescent BLCs (Fig. 1).

In the aging process, bone loss is caused not only by enhanced bone resorption activity but also by functional impairments of MSCs, which show a shift of lineage commitment to adipogenesis at the expense of osteogenesis [14] and a concomitant decreased self-renewal capacity [15]. This leads to an imbalance in bone tissue between bone mass and fat, finally increasing the risk of fractures [16]. Under normal conditions, several transcription factors control the commitment of MSC differentiation to osteogenesis or adipogenesis in a mutually exclusive and fine-tuned fashion [17]. Thus, it is well established that a sequential activation of CCAAT enhancer binding protein beta (CEBPβ), gamma (CEBPγ), alfa (CEBPα), and finally peroxisome proliferator activated receptor gamma (PPARγ) direct differentiation to adipogenesis [18, 19], whereas RUNX2 and Sp7 transcription factor (SP7) are master regulators of osteogenesis [20, 21]. In an aging scenario, it is known that there is an imbalance between the pro-adipogenic and pro-osteogenic transcription factors: for example, the expression of the adipogenic PPARγ [22] is highly upregulated in aged murine MSCs [16]. However, the upstream signaling pathways driving this pathological shift remain elusive and are under intense investigation. Here we will discuss the most recent/relevant pathological molecular findings which are upstream of this imbalanced commitment of MSCs due to aging. Several cell-intrinsic mechanisms have been recently shown to be implicated in biased cell fate choice of MSCs during aging.

### Transcription factors

Several lines of evidence have pointed to the dysregulation of the activity of transcription factors which play a role in skeletal development as a cause of the enhanced adipogenesis of bone residing MSCs.

MAF bZIP transcription factor (MAF) is a transcription factor involved in the regulation of the development and differentiation of many organs and tissues such as lens, kidney, liver, and immune system [23]. It has been identified as a transcription factor that mediates the age-related shift in MSC differentiation [24]. In murine MSCs, a gene expression signature has been identified which decreased in an age-dependent fashion. Among these genes, *Maf* was found to show increased expression during osteogenesis of normal MSCs, but decreased expression with increasing murine age. Bone formation in *Maf−/−* mice is severely impaired, as is the in vitro osteogenesis of MSCs isolated from these null mice. Mechanistically, it has been shown that MAF is a binding partner of RUNX2, and together these factors regulate gene expression in cooperatively promoting osteogenic differentiation. Conversely, MAF supresses adipogenesis by downregulating the expression of PPARγ.

Forkhead box P1 (FOXP1), a transcription factor which controls multiple biological processes [25], has been recently described to play an essential role in the fate choices of aged MSCs [26]. Li and co-workers [26] showed that *FOXP1*expression declines with age in both murine and human MSCs, and *Foxp1* ablation in mice resulted in age-dependent bone loss. Moreover, MSCs from *Foxp1* mutant mice showed an enhanced adipogenesis and hampered osteogenesis. Interestingly, when overexpressed in MSCs coming from aged patients, FOXP1 increased replication capacity in MSCs and potentiated their osteogenic differentiation. Thus, reduction of FOXP1 expression in old MSCs leads to an enhancement of adipogenesis at the expense of osteogenesis. The molecular explanation for these findings is that FOXP1inhibits adipocyte differentiation by interacting with CEBPβ and promotes osteogenic differentiation through repression of NOTCH signaling pathway activation.

Core-binding factor subunit beta (CBFβ) is a key co-factor of RUNX2, forming a complex with essential roles in skeletal development and osteoblast differentiation [27, 28]. Recent studies have revealed that *Cbfβ* expression is reduced in aged mice, which show a decrease in bone mineral density and an increase in marrow adiposity [29]. Moreover, *Cbfβ* gene deletion at different osteoblast lineage stages (MSCs, osteoprogenitors, and osteoblasts) leads to increased bone marrow adiposity and lower bone mass. Wu and co-workers [28, 29] showed that absence of Cbfβ favors adipogenesis and that Cbfβ plays a critical role in maintaining osteoblastic lineages. At the molecular level, to maintain osteoblast commitment, Cbfβ supresses adipogenesis by inhibiting adipogenic gene expression and enhancing Wnt/β-catenin signaling.

### MicroRNAs

MicroRNAs (miRNAs) are 20–25-nucleotide, single-stranded noncoding RNAs involved in the repression of the expression of target genes by either mRNA degradation or translational inhibition. They have been linked to the differentiation shift of aged-MSCs.

MicroRNA 196 (MIR196) has been shown to be up-regulated in human and murine MSCs in an age-dependent fashion, along with the decreased expression of one of its targets, homeobox B7 (*HOXB7*) [30]. MSCs overexpressing HOXB7 show increased cell proliferation, reduced cellular senescence, and enhanced osteogenic differentiation. Moreover, *HOXB7* has been recently identified as a downregulated gene in MSCs from aged donors, with higher differentially methylated regions in its promoter [31].

Expression of microRNA 188 (MIR188) was found to be remarkably up-regulated in aged mice and humans [32]. Mice lacking MIR188 showed a significant decrease of age-associated bone loss and fat accumulation in bone marrow. Conversely, transgenic mice with osteoprogenitors overexpressing MIR188 had greater age-associated bone loss and fat accumulation in bone marrow. MIR188 directly targets histone deacetylase 9 (*Hdac9)* and RPTOR independent companion of MTOR complex 2 (*Rictor*) mRNAs, which have a role in bone metabolism [33, 34].

MSCs from old mice have been reported to exhibit increased levels of tumor protein p53 (p53), which in turn block the transcription of the microRNA 17–92 (MIR17–92) cluster. In particular, microRNA 17 (MIR17) overexpression could enhance osteogenic differentiation in old murine MSCs by modulating the expression of *Smurf1*, which is known to regulate bone cell function [35]. Both microRNA 23a (MIR23a) and microRNA 23b (MIR23b) have been found to be downregulated in aged mice and humans and have been suggested to be involved in the pathological differentiation of MSCs in aging [36]. Overexpression of MIR23a and MIR23b enhances osteogenesis differentiation in MSCs, whereas their inhibition increases adipogenesis. MIR23a and MIR23b target the expression of transmembrane protein 64 (*Tmem64*), whose expression correlates inversely to MIR23a and MIR23b in aging and which has been described to regulate the shift in the lineage commitment of MSCs, boosting adipogenesis differentiation [37]. MicroRNA 218 (MIR218) is strongly up-regulated in osteoblasts from aged mice and directly targets and downregulates *Rictor* expression, a component of the mechanistic target of rapamycin complex 2 (mTORC2) [38]. Osteoblasts with downregulated levels of Rictor have reduced adhesion potential and survival [39] and less capacity to undergo the mineralization process.

### Autophagy

A hallmark of cellular aging is the decline in autophagy activity [1], a basic mechanism of degrading unnecessary or dysfunctional cell components such as proteins and damaged mitochondria. Recent work revealed that pharmacological stimulation of autophagy in aged MSCs by the use of the mTOR inhibitor rapamycin restores both the osteogenic differentiation capacity and proliferation of MSCs in vitro. Moreover, when administered in vivo, rapamycin restored bone mineral density of senile osteoporotic aged mice [40]. Restoring the autophagy process in aged organisms has been proposed as a future molecular pathway target for clinical treatment of osteoporosis.

### Alterations of the nuclear lamina

Bone tissue is especially affected in progeroid laminopathies, devastating diseases of premature aging, such as Hutchinson-Gilford progeria syndrome (HGPS), mandibuloacral dysplasia type A and B, restrictive dermopathy, atypical progeria syndrome, and atypical Werner syndrome [41]. A- and B-type lamins are intermediate filaments which polymerize at the nucleoplasmic side of the inner nuclear membrane to form the nuclear lamina. Although initially the nuclear lamina was thought to mainly provide mechanical stability to the nucleus, many studies over the past decade have implicated lamins in a plethora of functions: chromatin structure regulation, gene expression, intracellular signaling pathway modulation, and development [42]. Progeroid laminopathies, including systemic disorders and tissue-restricted diseases, are due to mutations in the Lamin A/C (*LMNA*) gene (which encodes both Lamin A and C proteins) or a defective posttranslational processing of Lamin A, giving rise to pathological accumulation of immature forms of Lamin A or to mutant Lamin A proteins (prelamin A or progerin, respectively) [43]. At the cellular level, an abnormal blebbing of the nuclear membrane is characteristic, which is considered a hallmark of these diseases. Remarkably, low amounts of prelamin and progerin have been detected in normal aging cells, thus reinforcing the possibility of a role in normal chronological aging as well [44].

Both animal and in vitro human cell culture models of progeroid laminopathies have successfully recapitulated the aging phenotypes that patients exhibit [45–48]. Interestingly, in vitro human cell models of HGPS have shown an increased differentiation of MSCs towards the osteoblastic lineage (increased alkaline phosphatase activity, an early marker of osteogenesis in MSCs [3], and increased expression of osteogenic genes), reflecting a “premature” osteogenesis in vitro [49, 50].

Moreover, aged human MSCs due to prelamin A accumulation show an altered secretome enriched in osteogenesis-related proteins. This secretome leads to aberrant paracrine signaling triggering accelerated early osteogenesis in normal MSCs [51], which show increased alkaline phosphatase activity and *Runx2* expression. Among the increased secreted factors, insulin like growth factor binding protein 7 (IGFBP7) was identified. RNA silencing experiments revealed an essential role for IGFBP7 for maintaining viability of MSCs during the first steps of osteogenesis in which MSCs and pre-osteoblasts proliferate actively, thus suggesting a role for IGFBP7 in regulating osteogenic differentiation [51, 52]. Moreover, sheets of human MSCs overexpressing IGFBP7 enhanced bone healing in a rat tibial osteotomy model [52]. Similar results were previously obtained in aged vascular smooth muscle cells (VSMCs) which accumulated prelamin A [44]. The authors found that under prelamin A accumulation, VSMCs secreted factors which enhanced osteogenesis in mesenchymal precursors. Taken together, these results support the idea that an “early” accelerated osteogenesis is a part of the aging process of bone, which is favored by pathological forms of Lamin A, contributing to an unbalanced homeostasis of bone tissue. However, these results seem to contradict the aforementioned shift commitment to adipogenesis in aging MSCs. One theory could be that this accelerated differentiation towards osteogenic lineages and increased secretion of osteogenesis-related proteins in MSCs is a kind of compensatory mechanism induced by prelamin A or progerin. It will be important to unravel whether this premature osteogenesis in prematurely aged MSCs due to accumulation of pathological forms of Lamin A is an efficient osteogenesis or whether it is dependent on the presence or not of pathological forms of Lamin A in MSCs.

Overall, these studies have clarified four mechanisms which seem to be crucial for the imbalanced differentiation of MSCs in aging: (1) the progressive downregulation of some key transcription factors essential for skeletal development; (2) the dysregulation of certain miRNAs with a functional role in bone homeostasis; (3) autophagy impairment; and (4) nuclear lamina alterations.

### Epigenetic modifications of DNA and reactive oxygen species

Epigenetic modifications of DNA and oxidative stress as a consequence of reactive oxygen species (ROS) accumulation due to aging processes seem to underlie the dysregulation of some of the above-mentioned mechanisms. In fact, several lines of evidence support a causative link between increased oxidative stress and the epigenetic changes observed in aging [53]. Therefore, it is known that MSC fate is determined by transcription factors that are regulated more specifically at the epigenetic level, where a specific chromatin configuration is conferred to control the expression of key transcription factors for differentiation. Recent work comparing the methylome of young aged human MSCs demonstrated that DNA methylation changes are associated with aging in MSCs, leading to a higher number of genes with decreased expression levels [31]. Li and co-workers point to the increased methylation of *FOXP1* promoter as a possible mechanism for the downregulation of the *FOXP1* expression in aging [26]. Regarding ROS, it has been shown that they can downregulate *MAF* expression in both mouse and human MSCs [5, 24] and upregulate the levels of MIR218, suggesting that enhanced ROS production in aging is the cause of the increase of MIR218 in aged osteoblasts [38].

### Cell-extrinsic factors

**Fig. 2:**
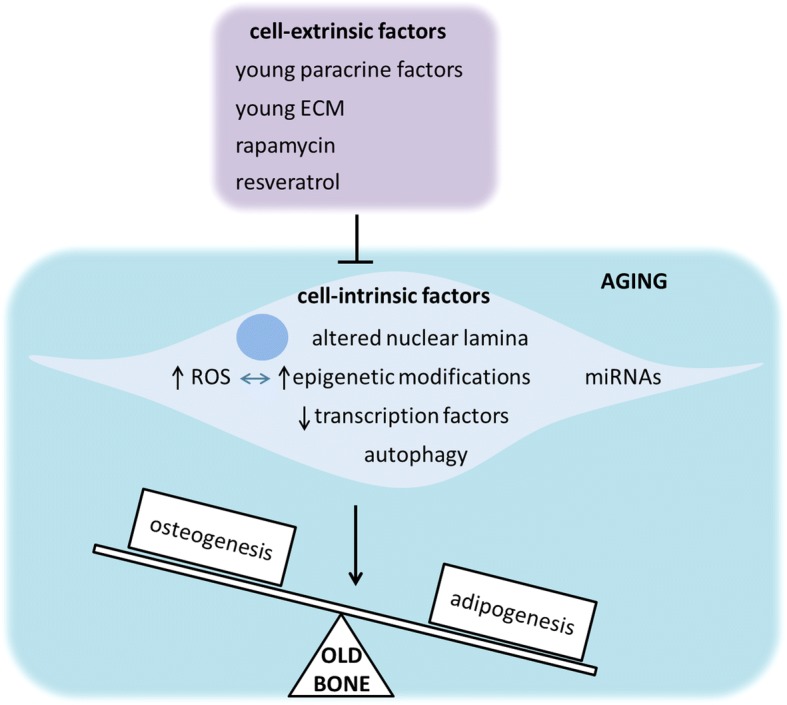
Age-related alterations of a number of cell-intrinsic factors can shift MSC differentiation to adipogenesis. Cell-extrinsic factors such as young paracrine factors and ECM, rapamycin, and resveratrol have been proven to restore osteogenic potential of aged MSCs through modulation of these impaired cell-intrinsic factors

Recently, a number of studies have explored the possibility that cell-extrinsic factors could restore the impaired osteogenesis of old MSCs by affecting either key osteogenic signaling pathways or the impaired autophagy of old MSCs (Fig. 2). Supporting this hypothesis, heterochronic parabiosis, that is, the exposure of aged mice to a young circulation, has been recently shown to enhance in vivo fracture repair and in vitro osteoblast differentiation in a manner similar to the effects of young bone marrow transplantation. The authors showed that this “rejuvenation” was governed by endogenous aged osteoblasts responding to a circulating young factor which modulated the Wnt/β-catenin pathway. Furthermore, this unidentified young factor was able to induce bone matrix deposition and mineralization of MSCs in vitro. To support this finding, the authors used conditioned media from young bone marrow aspirate stem cells (with mesenchymal and hematopoietic lineages), which could rescue the age-related decrease of osteogenesis of old MSCs in vitro. Conversely, conditioned media from old bone marrow aspirate stem cells was unable to alter the osteogenic potential of young MSCs [54]. Previous work demonstrated that MSCs from both young and old mice cultured in vitro in a young ECM, loaded into gelatine sponges, and then implanted into the dorsal surface of immunodeficient mice were capable of inducing bone formation in a similar fashion. The authors found that this restoration of osteogenic capacity of old MSCs could be due to the different molecular composition of young and old ECM. Indeed, old ECM exhibited a higher ratio of mineral to collagen than that observed in young MSCs [55]. Activation of autophagy by rapamycin can restore bone loss in aged mice and osteogenesis of MSCs possibly by regulating ROS levels [40]. Resveratrol, a polyphenolic compound found in red wine, has been found to promote in vivo bone formation and counteract bone loss in SAMP6 mice, a premature aging mouse model, and in naturally aging mice as well [56]. Moreover, resveratrol treatment in vitro rescued the osteogenic decline of MSCs from the aged mice mentioned above. The molecular mechanism responsible for this osteogenesis recovery involves the upregulation by resveratrol of the expression of mitofilin, a mitochondrial inner membrane protein which is downregulated in senescent MSCs from aged mice and plays a key role in mitochondrial morphological and functional homeostasis. Interestingly, resveratrol has been shown to improve bone mineral density from zinc metallopeptidase, STE24 (Zmpste24) knockout mice, a mouse laminopathy model of premature aging. Prelamin A accumulation in these mice, which show extreme loss of bone mineral density and are prone to fractures, hampered the normal interaction between Sirtuin 1 (SIRT1) and Lamin A, which was shown to be necessary for SIRT1 deacetylase function. The authors found that resveratrol increased SIRT1–Lamin A binding to increase SIRT1 deacetylase activity, suggesting that this mechanism was responsible for restoring osteogenesis of MSCs in premature aging mice and therefore alleviating the progeroid features of these mice [57].

## Conclusions

Age-related bone loss is driven in part by a decline in MSC proliferation and function and an increased commitment of these MSCs to adipogenic lineages. In the present review we have summarized the most recent cell-intrinsic molecular mechanisms underlying this pathological MSC differentiation and the cell-extrinsic factors that have been described to counteract this imbalanced differentiation. Understanding the molecular mechanisms governing the hampered osteogenesis of aged MSCs will be crucial for developing new bone anabolic treatments to promote bone formation.

## Abbreviations

BLC: Bone lining cell
CBFβ: Core-binding factor subunit beta
CEBPα: CCAAT enhancer binding protein alpha
CEBPβ: CCAAT enhancer binding protein beta
CEBPγ: CCAAT enhancer binding protein gamma
ECM: Extracellular matrix
FOXP1: Forkhead box P1
HDAC9: Histone deacetylase 9
HGPS: Hutchinson-Gilford progeria syndrome
HOXB7: Homeobox B7
IGFBP7: Insulin like growth factor binding protein 7
LMNA: Lamin A/C
MAF: MAF bZIP transcription factor
MIR17: MicroRNA 17
MIR17–92: MicroRNA 17–92
MIR188: MicroRNA 188
MIR196: MicroRNA 196
MIR218: MicroRNA 218
MIR23a: MicroRNA 23a
MIR23b: MicroRNA 23b
miRNA: MicroRNA
MSC: Mesenchymal stem cell
MTOR: Mechanistic target of rapamycin kinase
mTORC2: Mechanistic target of rapamycin complex 2
P53: Tumor protein p53
PPARγ: Peroxisome proliferator activated receptor gamma
RICTOR: RPTOR independent companion of MTOR complex 2
ROS: Reactive oxygen species
RUNX2: Runt related transcription factor 2
siRNA: Small interfering RNA ᅟ
SIRT1: Sirtuin1
TGFβ: Transforming growth factor beta 1
TMEM64: Transmembrane protein 64
VSMC: Vascular smooth muscle cell
Zmpste24: Zinc metallopeptidase, STE24
